# Development of a novel lipid metabolism-based risk score model in hepatocellular carcinoma patients

**DOI:** 10.1186/s12876-021-01638-3

**Published:** 2021-02-12

**Authors:** Wenjie Wang, Chen Zhang, Qihong Yu, Xichuan Zheng, Chuanzheng Yin, Xueke Yan, Gang Liu, Zifang Song

**Affiliations:** 1 Department of Hepatobiliary Surgery, Union Hospital, Tongji Medical College, Huazhong University of Science and Technology, 1277 Jiefang Avenue, Wuhan, 430022 China; 2 Hepatic Surgery Center, Tongji Hospital, Tongji Medical College, Huazhong University of Science and Technology, 1095 Jiefang Avenue, Wuhan, 430030 China; 3 Clinical Medical Research Center of Hepatic Surgery at Hubei Province, Wuhan, China; 4 School of Life Science and Technology, Huazhong University of Science and Technology, Wuhan, 430074 China

**Keywords:** Lipid metabolism, Hepatocellular carcinoma, Predicting model

## Abstract

**Background:**

Liver cancer is one of the most common malignancies worldwide. HCC (hepatocellular carcinoma) is the predominant pathological type of liver cancer, accounting for approximately 75–85 % of all liver cancers. Lipid metabolic reprogramming has emerged as an important feature of HCC. However, the influence of lipid metabolism-related gene expression in HCC patient prognosis remains unknown. In this study, we performed a comprehensive analysis of HCC gene expression data from TCGA (The Cancer Genome Atlas) to acquire further insight into the role of lipid metabolism-related genes in HCC patient prognosis.

**Methods:**

We analyzed the mRNA expression profiles of 424 HCC patients from the TCGA database. GSEA(Gene Set Enrichment Analysis) was performed to identify lipid metabolism-related gene sets associated with HCC. We performed univariate Cox regression and LASSO(least absolute shrinkage and selection operator) regression analyses to identify genes with prognostic value and develop a prognostic model, which was tested in a validation cohort. We performed Kaplan-Meier survival and ROC (receiver operating characteristic) analyses to evaluate the performance of the model.

**Results:**

We identified three lipid metabolism-related genes (*ME1*, *MED10*, *MED22*) with prognostic value in HCC and used them to calculate a risk score for each HCC patient. High-risk HCC patients exhibited a significantly lower survival rate than low-risk patients. Multivariate Cox regression analysis revealed that the 3-gene signature was an independent prognostic factor in HCC. Furthermore, the signature provided a highly accurate prediction of HCC patient prognosis.

**Conclusions:**

We identified three lipid-metabolism-related genes that are upregulated in HCC tissues and established a 3-gene signature-based risk model that can accurately predict HCC patient prognosis. Our findings support the strong links between lipid metabolism and HCC and may facilitate the development of new metabolism-targeted treatment approaches for HCC.

## Background

Liver cancer is one of the most common malignancies worldwide [1], with HCC being the most predominant pathological types of primary liver cancer [2]. The most common treatment methods for HCC are surgical resection and systemic, comprehensive treatment [3]. However, HCC is often diagnosed at late stages, leading to high rates of recurrence and metastasis after surgical resection, resistance to chemotherapy, and poor 5-year overall survival rate (< 12 %) [4, 5]. Common traditional clinicopathological characteristics, such as age, gender, and incision margin, cannot accurately predict the prognosis of HCC patients [6]. Therefore, the development of novel effective treatment methods and the identification of robust prognostic biomarkers are urgently needed.

Metabolic reprogramming is widely accepted as a hallmark of cancer. It involves aberrant alterations in various key metabolic pathways, including glycolysis and fatty acid, glutamine, and cholesterol metabolic pathways [7]. Metabolic reprogramming is essential for cancer cells to meet their high energy demands due to their rapid growth, as well as to adapt to changes in the tumor microenvironment [8]. Metabolic reprogramming is particularly important in liver cancer [9]. The liver is essential in maintaining the energy balance in our body and regulating lipid metabolism. Mounting evidence suggests that lipid metabolic reprogramming is strongly linked to cancer development and progression [10] and that alterations in lipid metabolism are common in HCC [11]. Thus, modulating lipid metabolic pathways may represent a promising treatment approach for HCC. Nevertheless, the effect of lipid metabolism-related gene expression in HCC patient prognosis remains unclear.

In this study, we performed GSEA to identify lipid metabolism gene sets that are significantly enriched in HCC patients. We also explored the role of lipid metabolism-related genes in HCC patient prognosis by combining mRNA expression profiles and clinical data from TCGA database. We identified three lipid metabolism-related genes that accurately predicted HCC patient prognosis. The findings presented herein strongly support that lipid metabolism has a promising prognostic and therapeutic value in HCC.

## Methods

### Gene expression data acquisition

Gene expression data, gene methylation profiles, and clinicopathological characteristics of HCC patients were obtained from TCGA [12]. The clinical information included survival time, survival status, gender, age, and tumor grade and stage; the samples of patients with missing clinical information were excluded. Data from 424 patients were analyzed in this study, and their clinical information was examined. Gene mutations in HCC patients were identified using cBioPortal (https://www.cbioportal.org/) [13].

### Human protein atlas (HPA)

The human protein atlas database (https://www.proteinatlas.org/) was used to obtain immunohistochemistry staining data. These data were used to assess the levels of different proteins in normal and tumor tissues.

### Enrichment analysis

GSEA was performed to identify gene sets significantly enriched in HCC patients [14]. The analysis was conducted using the GSEA v. 4.0.2 software from Broad Institute (http://www.broadinstitute.org/gsea). GSEA was carried out using a weighting method, and the number of random combinations was set to 1000; the minimum number of excluded genes was set to 1. The remaining parameters were set to default. False discovery rate (FDR) q-value < 0.05 was considered significant.

Functional enrichment analysis was performed using DAVID (The Database for Annotation, Visualization, and Integrated Discovery; https://david.ncifcrf.gov/) [15]. Enriched functions were visualized using SangerBox (http://sangerbox.com/Tool).

### Statistical analysis

Bioinformatic analyses were conducted using R version 3.6.1. We used the Wilcoxon rank-sum test to identify differentially expressed genes between HCC and normal tissues. Univariate Cox regression analysis was performed using the R survival package to identify genes with significant prognostic value (*p* < 0.05). LASSO regression was implemented by using the “glmnet” package to build a prognostic model. Combining regression coefficients and mRNA expression profiles, we established a formula to calculate a prognostic risk score: $risk\,score=\sum ({\text{Coefi}} \times {\text{Xi}})$. Coefi is the regression coefficient, and Xi represents the corresponding mRNA expression. A risk curve was plotted using R to evaluate the prognostic model. The differences in the overall survival of patients were assessed by using Kaplan-Meier analysis and log-rank method. The predictive performance of the model was evaluated using ROC analysis and the AUC (area under the curve) values.

## Results

### Identification of the lipid metabolism-related gene sets in HCC

**Fig. 1:**
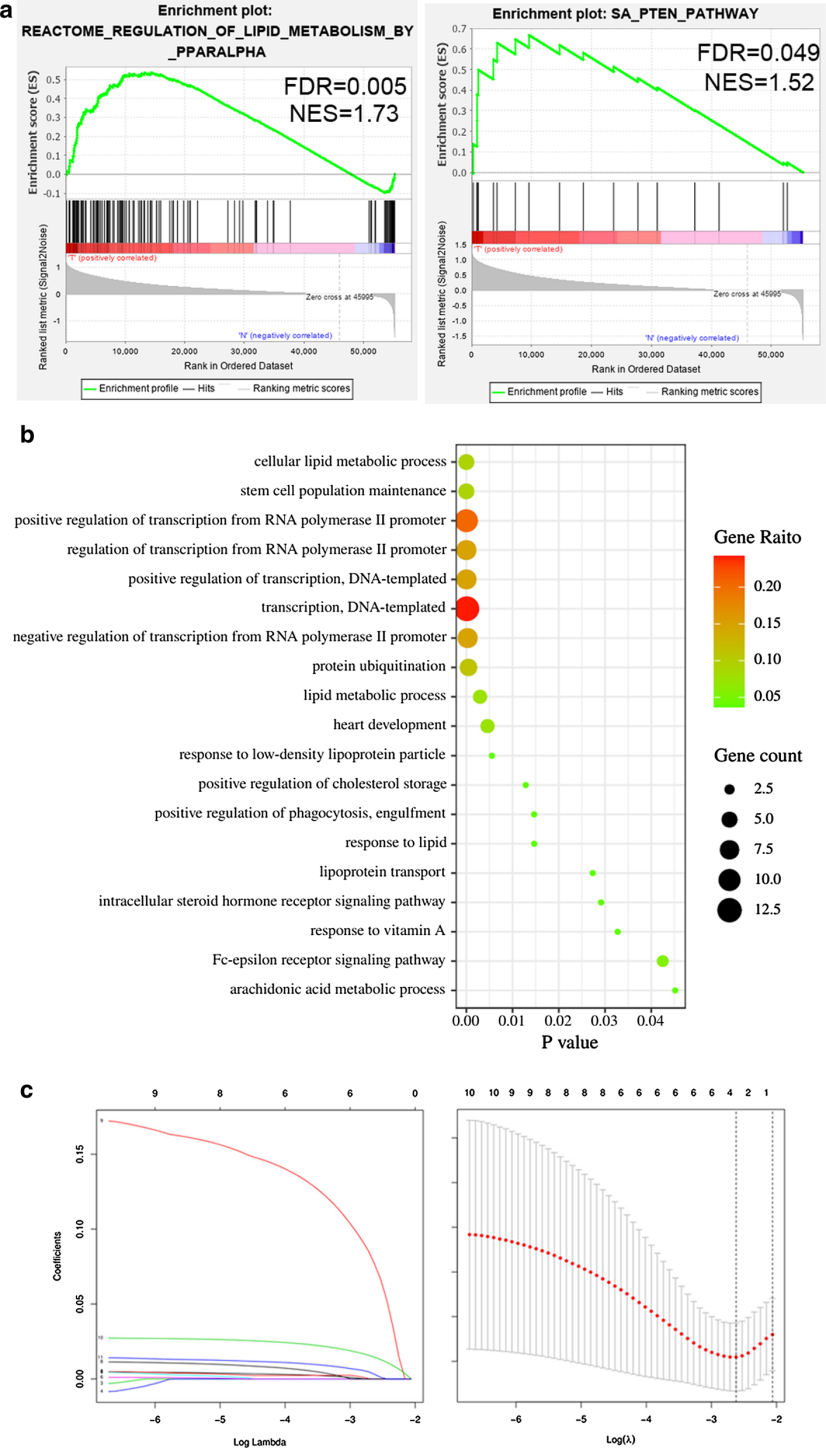
Enrichment analysis and LASSO regression analysis in HCC. **a** Gene set enrichment analysis of two lipid metabolism-related gene sets in HCC. **b** GO enrichment analysis of differentially expressed lipid metabolism-related genes in biological process. **c** Plots of regression coefficient using the LASSO model. On the left is the variation of LASSO coefficient with lambda for the 11 mRNAs from univariate Cox regression analysis. The first dotted line on the right represents the minimum values for logarithm Lambda according to the regression model

First, we identified nine gene sets associated with lipid metabolism by searching the keyword “lipid metabolism” in the MSigDB database. Subsequently, we assess for enrichment of these gene sets in HCC. GSEA results indicated that only two lipid metabolism-related gene sets were significantly enriched in HCC samples: “Reactome regulation of lipid metabolism by pparalpha” and “SA/PTEN pathway” (n = 163 genes; Fig. 1a). To investigate the expression level of these genes in HCC, we downloaded RNA-seq data from TCGA. We identified 35 lipid metabolism-related genes differently expressed between normal and tumor samples (*p* < 0.05, |logFC| > 1; Table 1). Next, we performed GO (gene ontology) enrichment analysis to get further insight into the function of these genes and confirmed their role in lipid metabolism (Fig. 1b).

**Table 1:** The detailed information of 35 mRNAs significantly up regulated in HCC patients

Up-regulated gene set		
mRNA	Ensembl ID	Description
TIAM2	ENSG00000146426	TIAM Rac1 associated GEF 2
MED25	ENSG00000104973	Mediator complex subunit 25
SHC1	ENSG00000160691	SHC adaptor protein 1
RXRB	ENSG00000204231	Retinoid X receptor beta
FADS1	ENSG00000149485	Fatty acid desaturase 1
AHRR	ENSG00000063438	Aryl-hydrocarbon receptor repressor
MAPK3	ENSG00000102882	Mitogen-activated protein kinase 3
TXNRD1	ENSG00000198431	Thioredoxin reductase 1
MED7	ENSG00000155868	Mediator complex subunit 7
NPAS2	ENSG00000170485	Neuronal PAS domain protein 2
NCOR2	ENSG00000196498	Nuclear receptor corepressor 2
TGS1	ENSG00000137574	Trimethylguanosine synthase 1
NCOA6	ENSG00000198646	Nuclear receptor coactivator 6
NRF1	ENSG00000106459	Nuclear respiratory factor 1
MED24	ENSG00000008838	Mediator complex subunit 24
MED15	ENSG00000099917	Mediator complex subunit 15
MED20	ENSG00000124641	Mediator complex subunit 20
TNFRSF21	ENSG00000146072	TNF receptor superfamily member 21
PPARG	ENSG00000132170	Peroxisome proliferator activated receptor gamma
MED22	ENSG00000148297	Mediator complex subunit 22
MED12	ENSG00000184634	Mediator complex subunit 12
PDPK1	ENSG00000140992	3-Phosphoinositide dependent protein kinase 1
CYP7A1	ENSG00000167910	Cytochrome P450 family 7 subfamily A member 1
ME1	ENSG00000065833	Malic enzyme 1
SIN3B	ENSG00000127511	SIN3 transcription regulator family member B
SREBF2	ENSG00000198911	Sterol regulatory element binding transcription factor 2
CD36	ENSG00000135218	CD36 molecule
MED10	ENSG00000133398	Mediator complex subunit 10
NFYA	ENSG00000001167	Nuclear transcription factor Y subunit alpha
CYP4A11	ENSG00000187048	Cytochrome P450 family 4 subfamily A member 11
PLIN2	ENSG00000147872	Perilipin 2
ACSL1	ENSG00000151726	Acyl-CoA synthetase long chain family member 1
GPS2	ENSG00000132522	G protein pathway suppressor 2
MED27	ENSG00000160563	Mediator complex subunit 27
SMARCD3	ENSG00000082014	SWI/SNF related, matrix associated, actin dependent regulator of chromatin, subfamily D, member 3

### Identification of lipid metabolism-related genes with prognostic value in HCC

To assess the relationship between the 35 lipid metabolism-related genes and HCC patients’ overall survival, we performed univariate Cox regression analysis. We identified 11 genes that were significantly associated with patient prognosis (Table 2). Ten of these 11 genes were associated with poor prognosis (hazard ratio [HR] > 1), and one gene (*CYP4A11*) predicted favorable outcomes (HR < 1). To selected the genes based on the minimum criterion, we conducted LASSO regression (Fig. 1c), which allows for the handling of variables even when there is collinearity between data [16]. Three lipid metabolism-related genes (*ME1*, *MED10*, *MED22*) were associated with patient survival.

**Table 2:** 11 mRNAs identified by univariate Cox analysis in HCC

mRNA	HR	HR.95L	HR.95H	coxPvalue
SHC1	1.4658	1.1424	1.8808	0.0026
MAPK3	1.5682	1.1715	2.0993	0.0025
NPAS2	1.5439	1.1773	2.0246	0.0017
MED15	1.7047	1.2296	2.3634	0.0014
MED20	1.4030	1.0303	1.9106	0.0316
CYP4A11	0.9051	0.8287	0.9885	0.0266
TNFRSF21	1.2054	1.0531	1.3798	0.0067
PPARG	1.2667	1.0569	1.5182	0.0105
MED22	1.8974	1.3858	2.5979	0.0001
ME1	1.2438	1.1009	1.4053	0.0005
MED10	1.7496	1.3406	2.2835	< 0.0001

### ***ME1***, ***MED10***, and***MED22***expression levels are elevated in HCC tissues

**Fig. 2:**
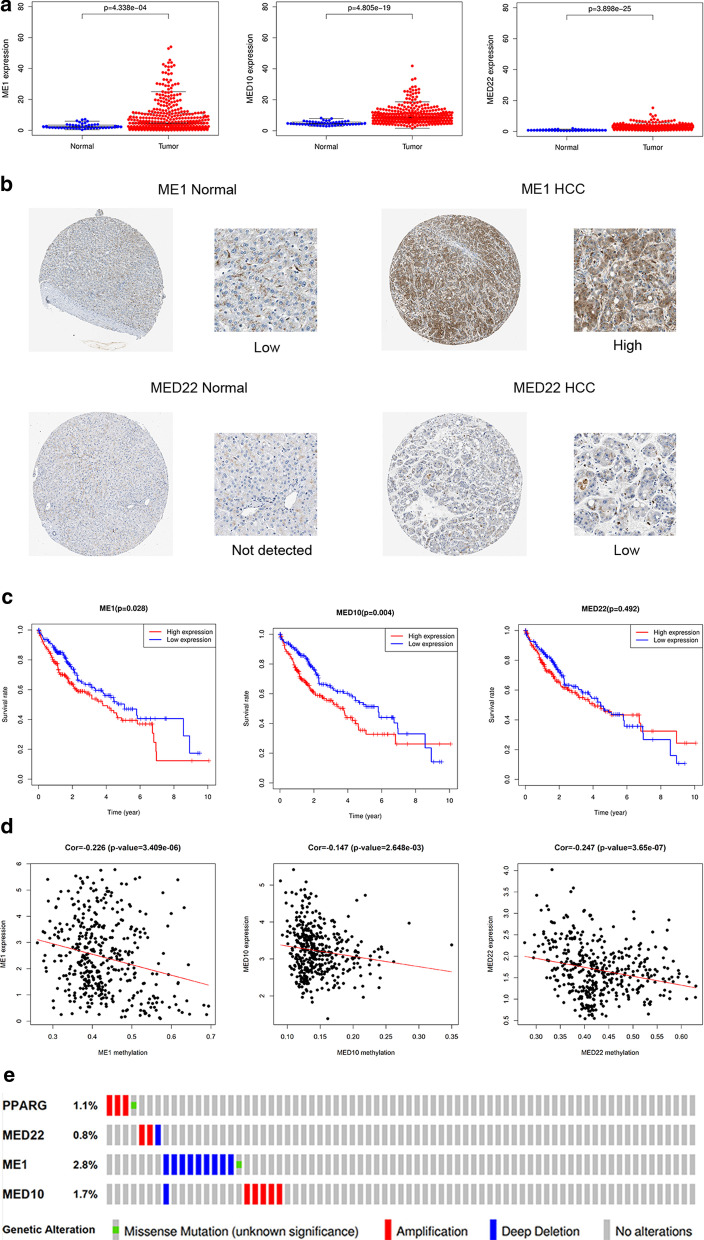
The expression level and epigenetic modification of three genes in HCC. **a** Differential expression analysis of three genes in mRNA level. **b** Representative immunohistochemistry staining of three proteins in normal tissue and HCC tissue from The Human Protein Atlas. **c** K-M analysis of three mRNAs. **d** Correlation between gene expression level and methylation degree. **e** Mutations of three genes in HCC

We assessed the expression levels of *ME1*, *MED10*, and *MED22* in HCC and normal tissues using data from TCGA and HPA and found that they were highly expressed in HCC tissues, both at the mRNA and protein levels (Fig. 2a, b). Based on the median expression of each gene, HCC patients were stratified into high-expression and low-expression groups. Survival analysis revealed that the expression level of each gene was associated with HCC patient survival (Fig. 2c).

To investigate the possible mechanism underlying *ME1*, *MED10*, and *MED22* upregulation in HCC, we analyzed the methylation levels of these genes in HCC patients. The mRNA levels of ME1, MED10, and MED22 were negatively correlated with their overall methylation levels (Fig. 2d), suggesting that their upregulation in HCC tissues might be due to a reduction in methylation levels. Furthermore, mutation analysis in HCC samples revealed that 0.8%, 2.8%, and 1.7% of patients had mutations in *MED22, ME1*, and *MED10*, respectively (Fig. 2e). Interestingly, most of the genetic alterations in these genes involved amplifications and deep deletions, suggested a potential role of gene mutations in ME1, MED10, and MED22 upregulation in HCC.

### Establishment and validation of a prognostic model

**Fig. 3:**
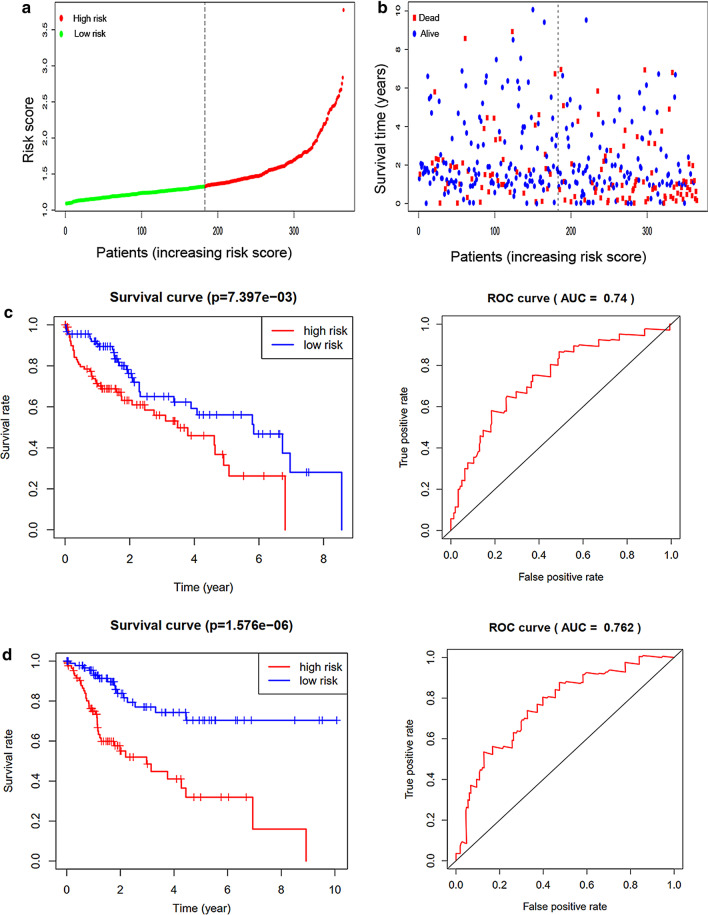
Construction and validation of a three mRNAs prognostic model. **a** HCC patients were divided into high-risk and low-risk groups according to their median risk scores. **b** Survival time and survival status of patients changed along with the value of risk score. **c** K-M analysis and ROC curve for training set. **d** K-M analysis and ROC curve for validation set

We randomly divided 370 HCC patients into a training set (n = 185) and a validation set (n = 185). Combining the regression coefficients of the three genes and their mRNA levels, we calculated the corresponding risk score for each patient [Risk score = (0.0144 × Expression of ME1) + (0.0030 × Expression of MED10) + (0.0763 × Expression of MED22)]. To construct a prognostic model based on these genes, we divided the patients in the training set into high-risk and low-risk groups according to their risk score (the median value was used as a cut-off; Fig. 3a). Interestingly, a high-risk score was associated with shorter survival time and increased mortality rate (Fig. 3b). Consistently, Kaplan-Meier analysis revealed a significant difference in overall survival between high-risk and low-risk patients, with a 5-year survival rate of 0.3 in the high-risk group and 0.6 in the low-risk group (Fig. 3c). ROC curve showed an AUC value of 0.74 (Fig. 3d), confirming the high prognostic value of our 3-gene signature in HCC.

We also verified the relation between clinical features and risk score by doing chi-square test (Table 3). The results revealed that age, grade and relative family cancer history significantly concerned the risk score of HCC patients.

**Table 3:** Clinical pathological parameters of patients and the chi-square test of the relation between clinical features and risk score

Clinical feature	N	Risk score		X^2^	*p*
		High risk n(%)	Low risk n(%)		
Age				66.60	< 0.001
> 65	276	65 (23.55 %)	211 (76.45 %)		
≤ 65	192	117 (60.94 %)	75 (39.06 %)		
Gender				0.38	0.54
Male	309	123 (39.81 %)	186 (60.19 %)		
Female	160	59 (36.88 %)	101 (63.13 %)		
Child pugh classification grade				2.41	0.12
A	271	103 (38.01 %)	168 (61.99 %)		
B–C	33	8 (24.24 %)	25 (75.76 %)		
Adjacent hepatic tissue inflammation				0.00	0.98
Yes	146	48 (32.88 %)	98 (67.12 %)		
No	162	53 (32.72 %)	109 (67.28 %)		
BMI				1.95	0.16
≥ 30	93	31 (33.33 %)	62 (66.67 %)		
< 30	324	134 (41.36 %)	190 (58.64 %)		
Cirrhosis				0.23	0.63
Yes	93	27 (29.03 %)	66 (70.97 %)		
No	119	31 (26.05 %)	88 (73.95 %)		
Grade				16.73	< 0.001
I–II	295	94 (31.86 %)	201 (68.14 %)		
III–IV	166	85 (51.20 %)	81 (48.80 %)		
Stage				2.60	0.11
I–II	306	118 (38.56 %)	188 (61.44 %)		
III–IV	112	53 (47.32 %)	59 (52.68 %)		
Barcelona clinic liver cancer				0.14	0.71
A–B	370	144 (38.92 %)	226 (61.08 %)		
C–D	31	11 (35.48 %)	20 (64.52 %)		
Neoadjuvant treatment				1.27	0.26
Yes	2	0 (0.00 %)	2 (100.00 %)		
No	467	182 (38.97 %)	285 (61.03 %)		
Radiation therapy				0.14	0.71
Yes	9	3 (33.33 %)	6 (66.67 %)		
No	372	147 (39.52 %)	225 (60.48 %)		
Person neoplasm cancer status				1.32	0.25
Tumor free	195	70 (35.90 %)	125 (64.10 %)		
With tumor	165	69 (41.82 %)	96 (58.18 %)		
Relative family cancer history				6.51	0.01
Yes	162	49 (30.25%)	113 (69.75 %)		
No	248	106 (42.74%)	142 (57.26 %)		

Subsequently, we tested our prognostic model in the validation set. Consistent with the findings in the training set, patients in the high-risk group had worse outcomes than those in the low-risk group (Fig. 3c). In the validation set, the prognostic model provided an AUC value of 0.762 (Fig. 3d), confirming that the 3-gene signature can accurately predict HCC patient prognosis.

### The 3-gene signature is an independent prognostic factor in HCC

**Fig. 4:**
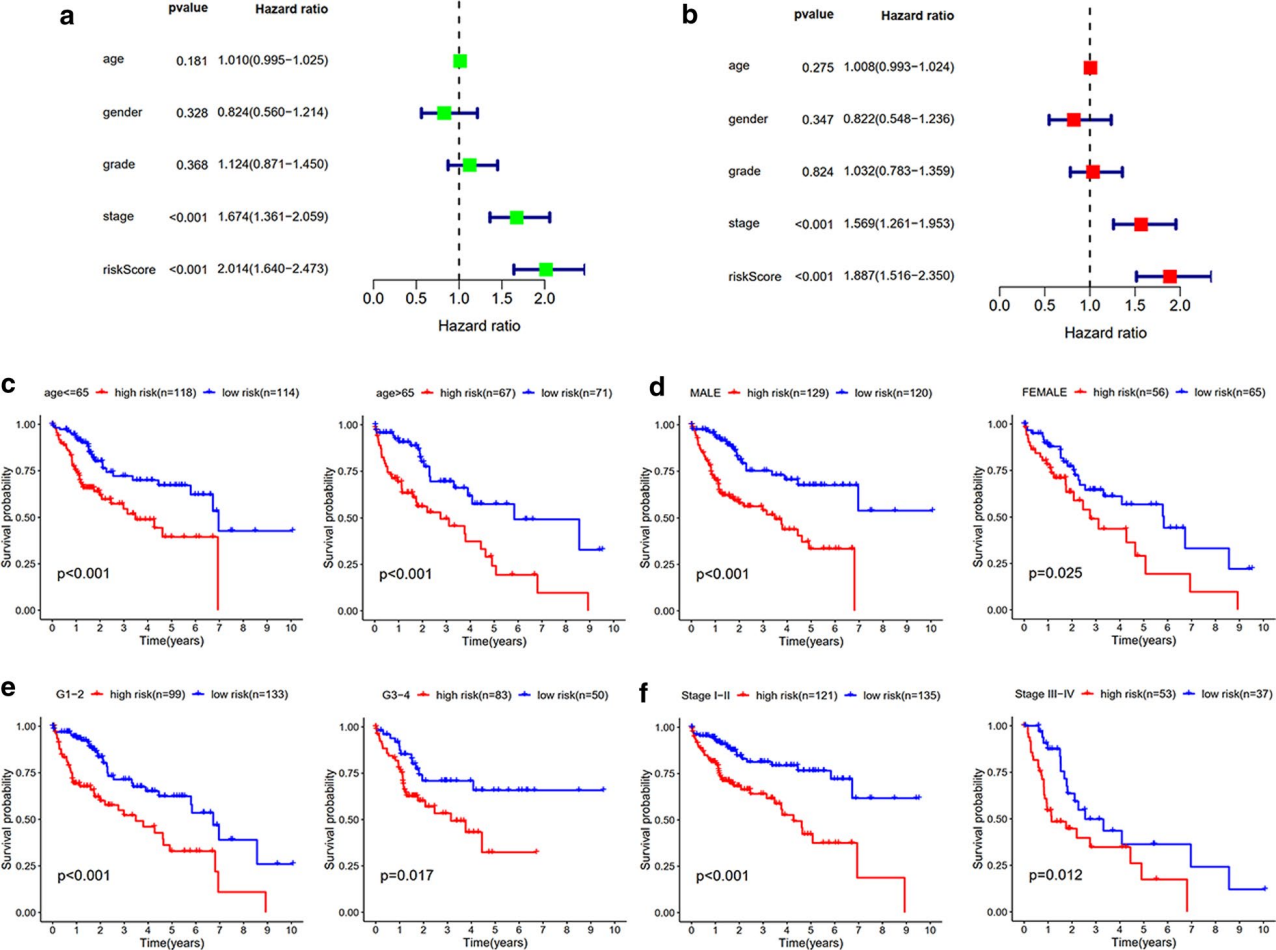
Independent prognosis analysis and stratified analysis of the three-mRNA signature. **a** Univariable COX regression analysis for clinical features. **b** Multivariable COX regression analysis for clinical features. **c** Stratified analysis for patients divided into age; **d** stratified analysis for patients divided into gender; **e** stratified analysis for patients divided into stage; (F) stratified analysis for patients divided into grade

To explore whether the risk score based on these three genes could be used as an independent prognostic factor, we performed univariate and multivariate cox regression analyses and found that the risk score was significantly associated with HCC patient prognosis (Fig. 4a, b). We also found that the tumor stage was an independent prognostic factor for poor prognosis.

To confirm the applicability of our prognostic model, we performed stratified survival analysis according to gender, age, tumor grade, and stage, and. The prognostic model was applied to the risk score of patients in each group. The survival rate of patients in the high-risk and low-risk groups was significantly different in each group [Fig. 4c–f].

## Discussion

HCC is the third cause of cancer-related deaths [17] and the sixth most common cancer worldwide, accounting for approximately 75–85 % of all liver cancers [18]. In recent years, metabolic reprogramming is widely regarded as a hallmark of liver cancer associated with rapid cell proliferation [19, 20]. Although the Warburg effect and glutamine metabolism alterations are common processes in the metabolic reprogramming of tumors [21, 22]. Mounting evidence also suggests that lipid metabolic reprogramming is essential for HCC development [23]. For instance, HCC cells typically exhibit enhanced *de novo* synthesis of fatty acids [24]. Lipid metabolism alterations also play a critical role in the adaptation of cancer cells to hypoxic conditions and maintenance of cancer stem cells [25]. When it comes to tumors and metabolism, biomarkers flash in our minds. Although there are many studies on lipid metabolism reprogramming in liver cancer, studies linking lipid metabolism with liver cancer prognosis are still limited. In this way, we tried to find out a lipid metabolism-related biomarkers for HCC patients.

In this study, we performed various bioinformatics analyses and revealed the role of lipid metabolism-related genes in HCC patient prognosis. Specifically, we identified two lipid metabolism-related gene sets (totaling 163 genes) significantly enriched in HCC. Developed by David Cox [26], Cox regression is widely used to compare the survival of two or more groups and evaluate the effect of factors on survival. In this way, we identified 11 lipid metabolism-related genes that were associated with patient survival by performing univariate Cox regression analysis. Used LASSO regression analysis, were eliminated 8 functionally similar mRNAs. We used the remaining 3 mRNAs (ME1, MED10, and MED22) to construct a prognostic model.

The use of risk scores can facilitate a more accurate prediction of patient prognosis and guide clinical decision making [27]. An overwhelming number of genes have been identified as potential prognostic biomarkers for cancer [28–30]. Consequently, appropriate mathematical models for patient risk assessment are required. Here, we calculated a risk score for each patient based on the expression levels of *ME1*, *MED10*, and *MED22* and their corresponding coefficients. Survival analysis revealed a significant difference in the survival rate between the high-risk and low-risk groups. The risk curve constructed based on this prognostic model also confirmed that the expression of these three mRNAs and the number of deaths increased with increasing risk values. Similar analyses in a validation cohort confirmed the prognostic accuracy of this model. Univariate and multivariate Cox regression analyses indicated that the risk score and tumor stage were significant and independent prognostic factors in HCC. Stratified survival analysis showed that the survival rate of patients in the high-risk and low-risk group differed significantly in the different subgroups, highlighting the feasibility of our prognostic model.

ME1, MED10, and MED22 levels were found to be elevated in HCC tissues, both at the mRNA level and protein levels. ME1 is a multifunctional enzyme converting malate to pyruvate and is associated with lipid metabolism [31]. The beta-oxidation of fatty acid chains to produce energy requires acetyl-CoA [32]. In the meantime, a malic enzyme is needed for tricarboxylate shuttle so that acetyl-CoA can pass through the mitochondria into the cytoplasm [33]. The role of ME1 in cancer has previously been reported [34, 35]. It can induce epithelial-mesenchymal transition in a ROS (reactive oxygen species)-dependent manner [36]. MED10 and MED22 are both subunits of the Mediator complex, an evolutionary conserved multiprotein complex consisting of approximately 30 subunits. The Mediator complex functions as a transcriptional coactivator in eukaryotes [37, 38], regulating the expression of most RNA polymerase II-transcribed genes [39]. Understanding the function of the Mediator complex in cancer is critical, as it may guide the development of novel anti-cancer therapies [40]. However, the role of the Mediator complex in HCC merits further investigation.

Epigenetic modifiers are promising targets for the treatment of HCC, as they play an essential role in regulating HCC cell proliferation and metastasis [41]. DNA methylation is a critical epigenetic regulation mechanism, playing a vital role in tumor development by altering the expression of various tumor-associated genes [42]. Gene mutations are key to tumor development and progression [43]. In this study, we identified varying degrees of mutations in *ME1*, *MED10*, and *MED22* in HCC patients. We also found that their expression levels were negatively correlated with their gene methylation levels. These data suggest that the mutations and aberrant gene methylation in *ME1*, *MED10*, and *MED22* in HCC tissues might contribute to their upregulation in HCC. Therefore, a more in-depth understanding of the epigenetic changes in these genes may facilitate the development of targeted therapies for HCC.

## Conclusions

We identified three lipid-metabolism-related genes (*ME1*, *MED10*, and *MED22*) that are upregulated in HCC tissues, and established a 3-gene signature-based risk model that can accurately predict HCC patient prognosis. Notably, HCC patients with high-risk scores are more likely to experience worse outcomes than low-risk patients. We also provide preliminary data on the possible mechanisms underlying the pro-tumorigenic effects of these genes in HCC. The findings of this study support the strong link between lipid metabolism and HCC progression and may guide the development of novel metabolism-targeted treatment approaches for HCC.

## Data Availability

The gene expression datasets, gene methylation profiles, and clinicopathological characteristics of HCC patients were obtained from the cancer genome atlas database (https://tcga-data.nci.nih.gov/tcga/). The immunohistochemistry staining data was obtained from the human protein atlas database (https://www.proteinatlas.org/). Gene mutations in HCC patients were obtained from cBioPortal database (https://www.cbioportal.org/). Detailed information about 35 mRNAs in Table 1 was obtained from the human gene database (https://www.genecards.org/).

## Abbreviations

HCC: Hepatocellular carcinoma
TCGA: The Cancer Genome Atlas
GSEA: Gene set enrichment analysis
ROC: Receiver operating characteristic
LASSO: Least absolute shrinkage and selection operator
AUC: Area under the curve
GO: Gene ontology
DAVID: The Database for Annotation, Visualization, and Integrated Discovery
ROS: Reactive oxygen species
